# A U.S. Isolate of *Theileria orientalis* Ikeda Is Not Transstadially Transmitted to Cattle by *Rhipicephalus microplus*

**DOI:** 10.3390/pathogens12040559

**Published:** 2023-04-05

**Authors:** Cynthia K. Onzere, David R. Herndon, Amany Hassan, Kennan Oyen, Karen C. Poh, Glen A. Scoles, Lindsay M. Fry

**Affiliations:** 1 Department of Veterinary Microbiology and Pathology, Washington State University, Pullman, WA 99164, USA; cynthia.onzere@wsu.edu (C.K.O.); amany.hassan@wsu.edu (A.H.); kennan.oyen@usda.gov (K.O.); karen.poh@usda.gov (K.C.P.); 2 Animal Disease Research Unit, USDA-ARS, Pullman, WA 99164, USA; david.herndon@usda.gov; 3 Department of Animal Medicine, The Faculty of Veterinary Medicine, The University of Alexandria, Alexandria 21944, Egypt; 4 United States Department of Agriculture, Agricultural Research Service, Invasive Insect Biocontrol and Behavior Laboratory, Beltsville, MD 20705, USA; glen.scoles@usda.gov

**Keywords:** *Theileria orientalis*, cattle fever tick, *Rhipicephalus microplus*, vector, competence, transmission

## Abstract

*Theileria orientalis* Ikeda has caused an epidemic of bovine anemia and abortion across several U.S. states. This apicomplexan hemoparasite is transmitted by *Haemaphysalis longicornis* ticks; however, it is unknown if other North American ticks are competent vectors. Since the disease movement is largely determined by the host tick range(s), the prediction of the *T. orientalis* spread among U.S. cattle populations requires determination of additional competent tick vectors. Although *Rhipicephalus microplus* has mostly been eradicated from the U.S., outbreaks in populations occur frequently, and the U.S. remains at risk for reintroduction. Since *R. microplus* is a vector of *Theileria equi* and *T. orientalis* DNA has been detected in *R. microplus*, the goal of this study was to determine whether *R. microplus* is a competent vector of *T. orientalis*. Larval *R. microplus* were applied to a splenectomized, *T. orientalis* Ikeda-infected calf for parasite acquisition, removed as molted adults, and applied to two *T. orientalis* naïve, splenectomized calves for transmission. After 60 days, the naïve calves remained negative for *T. orientalis* by PCR and cytology. Additionally, *T. orientalis* was not detected in the salivary glands or larval progeny of acquisition-fed adults. These data suggest that *R. microplus* is not a competent vector of the U.S. *T. orientalis* Ikeda isolate.

## 1. Introduction

*Theileria orientalis* is a hemoprotozoan parasite in the phylum Apicomplexa [1]. The infection of cattle with the parasite can cause anemia, weakness, reduced milk production, reduced weight gain, abortion, and occasionally death [2,3,4,5,6,7,8]. Eleven genotypes of *T. orientalis* have been identified [1]. Of those, the most commonly encountered genotypes are Buffeli, Chitose, and Ikeda [1,9,10,11]. While Buffeli is generally clinically benign, Chitose and Ikeda tend to be more virulent, resulting in the increased morbidity and mortality of affected cattle [2,10]. Although small, transient outbreaks of *T. orientalis* Buffeli occurred sporadically in the United States (U.S.) before 2017 [12,13], *T. orientalis* Ikeda has since become endemic in Virginia [8,11,14,15] and has been detected in seven U.S. states to date [16]. In one recent study, up to 20% of the cattle tested at Virginia sale barns were positive for *T. orientalis* [11]. No vaccine is yet available to prevent disease, and there are no approved treatment options for affected cattle [2]. Since cattle that survive acute infection with *T. orientalis* become persistent, asymptomatic carriers [2], the continued spread of the parasite throughout the U.S. is possible as cattle are moved and the range of competent tick vectors expands.

Vector-competent ticks are the definitive hosts of *T. orientalis*, which spreads primarily by tick transmission [12,17]. In the U.S. and much of Asia, Australia, and New Zealand, the Asian longhorned tick, *Haemaphysalis longicornis*, is the primary vector of *T. orientalis* [2,17,18,19]. A large population of *H. longicornis* was detected in New Jersey in 2017 [20], and has since spread throughout eighteen states, including Virginia [15], West Virginia, North Carolina, Tennessee, Kentucky, Georgia, South Carolina, Missouri, Arkansas, Ohio, New York [21], New Jersey, Pennsylvania, Maryland, Connecticut, Delaware, Massachusetts, and Rhode Island [22,23]. Few other *Haemaphysalis* sp. ticks, including *H. bancrofti* and *H. humerosa*, are also known to be competent vectors of *T. orientalis* [2], and the parasite has also been transmitted iatrogenically and via mechanical insect vectors, including lice and biting flies [24,25].

The spread of tick-borne pathogens is largely dependent upon the geographic range of their competent tick vectors [26]. Thus, the accurate prediction of how *T. orientalis* will spread throughout U.S. cattle populations is dependent on determining whether other native tick species can also serve as competent vectors of the parasite. The cattle fever tick, *Rhipicephalus microplus*, is a major concern to U.S. cattle and equine producers due to its spread of hemoparasitic pathogens to cattle and horses, namely *Babesia bovis* and *Theileria equi* [27,28,29,30]. Although largely eradicated from the U.S. [31], this tick is still regularly encountered in buffer quarantine zones along the U.S.-Mexico border in Texas, and occasionally invades beyond the quarantine zone there [31]. With approximately 13% of all U.S. beef cattle raised in Texas [32], the determination of the vector potential of *R. microplus* for *T. orientalis* is critically important, especially considering that cattle persistently infected with *T. orientalis* could be transported to a region with an outbreak population of cattle fever ticks.

Since *R. microplus* is a competent vector of the related Apicomplexan parasites, *Theileria equi, Babesia caballi*, and *Babesia bovis* [29,30], and previous studies reported the detection of *T. orientalis* DNA in tissues of *R. microplus* ticks in *T. orientalis*-endemic areas [33,34,35,36,37,38], the goal of the present study was to determine whether *R. microplus* ticks are competent vectors of the U.S. isolate of *T. orientalis* Ikeda. The current study reports the outcome and implications of a controlled *T. orientalis* acquisition and transmission study using Holstein calves and colonized *R. microplus* ticks.

## 2. Materials and Methods

### 2.1. Cattle

In total, three 2–3-month-old Holstein calves obtained from local dairies were utilized in this study. Calves 1 and 2 were steers and Calf 3 was a freemartin. Prior to the study, all calves were splenectomized under the supervision of a board-certified agricultural animal internist at Washington State Veterinary Teaching Hospital and subsequently rested for 4–8 weeks to allow for full recovery. At the onset of the study, the calves were confirmed as healthy based on a physical exam, complete blood count (CBC), and serum chemistry panel, and were negative for *T. orientalis* via PCR for the *T. orientalis* Ikeda major piroplasm surface protein (MPSP, Section 2.3). Calf 1 was inoculated with *T. orientalis* Ikeda blood stabilate (below) and used for tick acquisition feeding. Calves 2 and 3 were subsequently utilized for tick transmission feeding. These animal experiments were approved by the University of Idaho Institutional Animal Care and Use Committees, Protocol 2021-37.

### 2.2. Infection of Calf 1 with Theileria orientalis Stabilate

Calf 1 was intravenously inoculated with 12 mL of cryopreserved *T. orientalis* Ikeda-infected erythrocyte stabilate composed of erythrocytes from 3 previously infected animals: 8.4 mL from stabilate batch 1804/3-2-22, parasitemia 2.5%; 2.4 mL from stabilate batch 1697/5-12-20, parasitemia 0.7%; and 1.2 mL from stabilate batch 1726/7-2-20, parasitemia 0.48%. All stabilates were prepared as previously described [17] and stored in liquid nitrogen until use. The stabilates were thawed rapidly, mixed with an autologous serum, and injected slowly into the jugular vein. Following inoculation, the calf was monitored for signs of anaphylaxis for 30 min.

Following inoculation, the attitude, appetite, rectal temperature, pulse, and respiratory rate were assessed daily, and the CBC and chemistry panel were assessed weekly. *T. orientalis* PCRs (Section 2.3) were conducted every 2–7 days. The packed cell volume (PCV) was assessed every 48 h. The percent parasitized erythrocytes (PPE) was determined every 48 h via an evaluation of the Diff-Quik-stained blood smears using the following equation: ((Total parasites in 5 fields)/(Erythrocyte count in ¼ of a field × 20)) × 100. At the end of the experiment, the calf was euthanized via an intravenous administration of sodium pentobarbital (Fatal Plus^®^, Vortech Pharmaceuticals, Dearborn, MI, USA).

### 2.3. Theileria orientalis DNA Isolation

For both cattle and ticks, the *T. orientalis* infection status was assessed via PCRs for *T. orientalis* MPSP as described in [8,17,39], with minor modifications. Briefly, the DNA was isolated from the whole bovine blood, macerated tick salivary gland tissues, or macerated larval ticks using the DNeasy Blood and Tissue kit (Qiagen, Hilden, Germany), in accordance with the manufacturer’s instructions. DNA was eluted in 100 µL of the elution buffer and the concentrations were determined using the DeNovix DS-11 series spectrophotometer/fluorometer (Denovix^®^, DeNovix Inc., Wilmington, DE, USA).

### 2.4. Theileria orientalis PCR

Following DNA isolation, a PCR master mix consisting of 22.5 µL of the AccuPrime™ Pfx SuperMix (Invitrogen™, Waltham, MA, USA), 2 µL of DNA at 20–100 ng/µL, and 1.5 µL at 10 µM each of forward (5′-CTTTGCCTAGGATACTTCCT-3′) and reverse (5′-ACGGCAAGTGGTGAGAACT-3′) primers was prepared. Conventional PCR was performed at the following thermal cycling conditions: initial denaturation at 95 °C for 5 min, followed by 35 cycles of denaturation at 95 °C for 15 s, annealing at 57 °C for 15 s, and extension at 68 °C for 1 min. DNA from a blood sample previously confirmed to be positive for *T. orientalis* Ikeda by microscopy and PCR was used as a positive control, and the UltraPure™ DNase/RNase-free distilled water (Thermo Fisher Scientific, Waltham, MA, USA) was used as a template in the negative control. In total, 10 µL of the PCR product was loaded onto the wells of a 1% agarose gel for visualization of the PCR product at the expected size of 776 base pairs. For assessment of bovine blood, a single aliquot of extracted DNA was assessed at each timepoint. For the tick salivary gland assessment, single aliquots of extracted DNA from the salivary glands of 15 stimulation-fed adult ticks were assessed from each group of ticks. For the assessment of larval ticks, 10–12 aliquots of extracted DNA from groups of ~20,000 macerated larvae were assessed.

### 2.5. Acquisition Feeding of Rhipicephalus microplus Ticks on Calf 1

The ticks utilized for the study were from a colony of the La Minita *R. microplus* strain, derived from an outbreak tick population in Star County, Texas in 1996 [40]. The colony has been maintained continuously (3–4 generations per year) at the Animal Disease Research Unit tick lab without the addition of new field-collected ticks since 1999, when it was acquired from Texas [40].

For acquisition, 2 groups of larval *R. microplus* ticks were applied to Calf 1, each comprised larvae hatched from 0.25 g of eggs (approximately 5000 ticks). The ticks were applied beneath separate, 8.5″ × 8″ cloth patches adhered to the shaved skin on the back of the calf with hip tag cement. To ensure the ticks were exposed to blood stages *T. orientalis*, the first group of larvae (Group 1) was applied 53 days after *T. orientalis* inoculation (33 days after the calf became PCR-positive for *T. orientalis* and 11 days after organisms were first detectable via blood smear cytology) and the second group (Group 2) was applied 13 days later (66 days post-*T. orientalis* inoculation).

### 2.6. Transmission Feeding of Rhipicephalus microplus Ticks on Calves 2 and 3

Tick feeding patches were applied to the backs of Calves 2 and 3, as described in Section 2.4. Approximately 1000 freshly molted adult *R. microplus* ticks from Group 1 were removed from Calf 1 16 days after application using forceps to gently extract freshly molted adults from previous exuviae (which remained attached to the host) and transferring them to a feeding patch on Calf 2. Approximately 1000 freshly molted adult *R. microplus* ticks from Group 2 were similarly removed 17 days after application and transferred to a feeding patch on Calf 3. These ticks were allowed to attach and feed to repletion, and replete females were subsequently collected for egg-laying. To assess whether the ticks had acquired *T. orientalis* from Calf 1, a subset of adult male and female ticks from each group (30 ticks total, 15 per group) were collected after 4–5 days of feeding (sufficient to allow salivary gland development and parasite replication) and the salivary glands were harvested, processed, and analyzed via *T. orientalis* MPSP PCR. as described in Section 2.2.

Following tick application, Calves 2 and 3 were monitored via a physical exam, CBC, chemistry panel, PCV analysis, peripheral blood PCR, and blood smear cytology for 60 days in the same manner as Calf 1, as described in Section 2.3. A sixty-day cut-off was three times the duration it took Calf 1 to become PCR-positive for *T. orientalis* following intravenous inoculation, which is less efficient than tick transmission. In previous studies, the calves infected via *H. longicornis* tick feeding became PCR-positive for *T. orientalis* on day 14 post-tick application [17]. Given these data, a 60-day window is likely sufficient for the development of patent infection. After 60 days, the calves were euthanized via the intravenous administration of sodium pentobarbital (Fatal Plus^®^, Vortech Pharmaceuticals, Dearborn, MI, USA).

### 2.7. Assessment of Progeny Larval Ticks for Theileria orientalis Infection

Eggs laid by replete adult female ticks from Groups 1 and 2 were incubated at 26 °C and allowed to hatch. In total, 2 g (~40,000) of progeny larvae from female ticks in each group were macerated as follows: 10 mL of sterile 1 × phosphate-buffered saline (PBS) (Thermo Fisher Scientific, Waltham, MA, USA) was added to the larvae from each group, the tick mixtures transferred to separate 15 mL Ten Broeck type tissue grinders (Thomas Scientific, Swedesboro, NJ, USA), and the ticks were macerated until the issues were completely homogenized. The homogenized tissue was then transferred to separate 25 mL Falcon^®^ conical centrifuge tubes (Corning Life Sciences, Corning, NY, USA) and centrifuged at 1800× *g* for 10 min. The supernatants were collected, vortexed, distributed in 2 mL aliquots, and frozen until ready for use. DNA extraction and *T. orientalis* MPSP PCRs were subsequently performed, as described in Section 2.2.

## 3. Results

### 3.1. Theileria orientalis Infection of Calf 1

**Figure 1:**
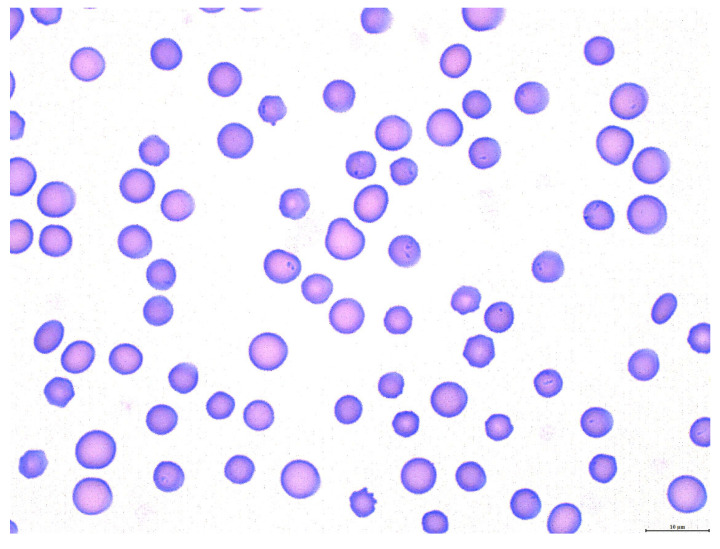
Photomicrograph of representative blood smear from Calf 1, day 111 post-inoculation with *T. orientalis*. Occasional erythrocytes contain 1–2, intracytoplasmic, 1–2.5 µm × 0.5 µm piroplasms, consistent with the *T. orientalis* merozoite stage. Scale bar: 10 µm.

**Figure 2:**
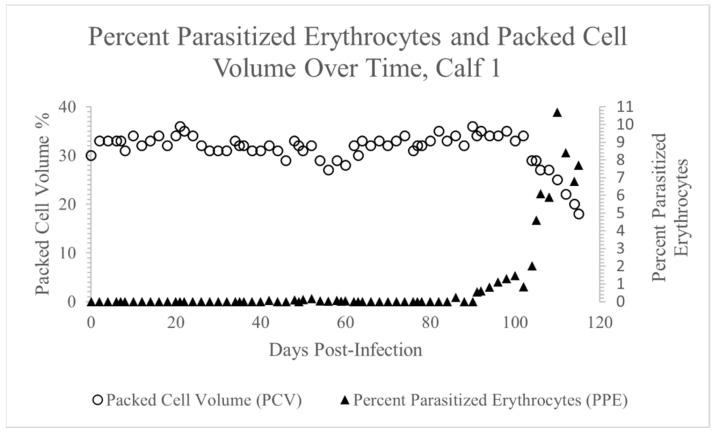
PPE and PCV over time, Calf 1. Piroplasms were first detected on day 42 post-inoculation, and from that point to the end of the experiment, PPE ranged from 0% to 10.7%. The PCV did not decline significantly until day 104, when it went into decline, reaching a nadir of 18% on day 115 post-inoculation.

*Theileria orientalis* was first detected via PCR in the peripheral blood of Calf 1 twenty days post-inoculation (Table 1). While *T. orientalis* was detected in the peripheral blood of this calf via PCR for most of the remaining experimental timeline, there were scattered days during weeks 3, 5, and 11 when the parasitemia temporarily dropped below the level of PCR detection (indicated by +/−, Table 1). Piroplasms were intermittently detected via blood smear cytology (Figure 1) from day 42 post-inoculation onward, and the PPE ranged from 0.025% to 10.7% (Figure 2). The PCV remained stable until day 104, when it began to steadily decline, reaching a nadir of 18% (12% decline from pre-infection PCV) on day 115 post-inoculation (Figure 2). Apart from anemia, the complete blood counts were normal throughout the experiment. Terminally, mild hypoproteinemia and hyperbilirubinemia were noted on serum chemistry panels, and the calf exhibited mild tachypnea, depression, and lethargy secondary to anemia. As in previous experiments [17], the calf never became febrile.

### 3.2. Rhipicephalus microplus Acquisition Feed on Calf 1

The first group of ~5000 *R. microplus* larvae were applied to Calf 1 on day 53 post-inoculation and ~1000 removed sixteen days later as freshly molted adults. During this time-period, Calf 1 was PCR-positive for *T. orientalis* Ikeda via PCR and exhibited PPE of 0.025–0.1%. The second group of ~5000 *R. microplus* larvae were applied to Calf 1 on day 66 post-inoculation and ~1000 removed seventeen days later as freshly molted adults. During this time-period Calf 1 was intermittently PCR-positive for *T. orientalis* and exhibited a PPE of 0–0.04%.

Following removal, the ticks were applied to Calves 2 (Group 1) and 3 (Group 2) and allowed to feed. After a brief (4–5 day) period of feeding, a subset of male and female ticks from each batch were removed, dissected, and their salivary glands assessed for *T. orientalis* via PCR. *T. orientalis* was not detected in the salivary glands of any of the fifteen assayed ticks from each group (Table 2). This finding supports the conclusion that *R. microplus* ticks did not acquire *T. orientalis* while feeding on Calf 1 in this experiment.

### 3.3. Failure of Adult R. microplus Ticks to Transmit T. orientalis to Calves 2 and 3

**Figure 3:**
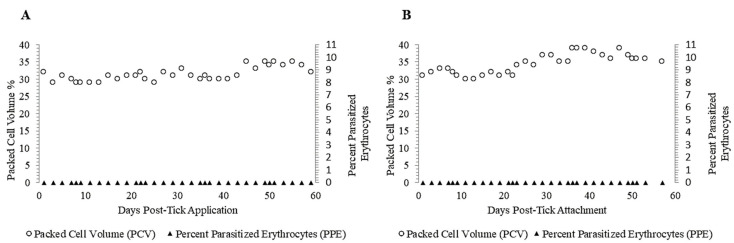
PPE and PCV over time, Calves 2 (**A**) and 3 (**B**). In both calves, no parasites were detected during the 60-day monitoring period post-tick application, and the PCV remained within the normal range for the entire experiment.

Following tick application, Calves 2 and 3 were monitored for evidence of *T. orientalis*-infection for 60 days. *T. orientalis* was not detected in the peripheral blood of either calf during that time-period via PCR (Table 1) or blood smear cytology (Figure 3A,B), consistent with lack of successful transmission of *T. orientalis* by *R. microplus* ticks in this experiment. Neither calf developed fever, anemia (Figure 3A,B), or any other abnormalities within the parameters assessed via physical exam, complete blood count or serum chemistry panel during this time-period.

### 3.4. T. orientalis Is Not Detected in Larval Progeny of R. microplus Fed on an Infected Calf

Since some Apicomplexan parasites, such as *B. bovis*, are transmitted transovarially by *R. microplus* ticks [41], ~40,000 progeny larval ticks of adults from both Groups 1 and 2 were assessed for *T. orientalis* via PCR. *Theileria orientalis* was not detected in progeny larvae from either tick group (Table 2).

## 4. Discussion

The results of this study suggest that *R. microplus* ticks are not competent vectors of the U.S. isolate of *T. orientalis* Ikeda via transstadial transmission. Larval stage *R. microplus* was applied to a *T. orientalis* Ikeda-infected calf and fed until adulthood. Once molted to adults, but before beginning to feed as adults, the ticks were removed and permitted to feed to repletion on two naïve, splenectomized calves to assess transstadial transmission. *T. orientalis* was not detected in the salivary glands of the adult *R. microplus* ticks after 4–5 days of transmission feeding, nor was it detected in progeny larvae of replete acquisition-fed adults. Furthermore, the two splenectomized, transmission-fed calves remained negative for *T. orientalis* Ikeda by both PCR and blood smear cytology at day 60 post-tick application and did not develop clinical signs consistent with infection.

Both *Theileria* sp. and *Babesia* sp. are apicomplexan hemoparasites that belong to the order Piroplasmida [42], and, although *R. microplus* ticks efficiently transmit *B. bovis* and *T. equi* [27,30,41,43,44], our study provides no evidence that they are vectors of *T. orientalis* Ikeda. This result may be related to the unique life history of *R. microplus* as a one-host tick. For *R. microplus* to be considered a competent vector of *T. orientalis* Ikeda, transmission must occur via at least one of two possible mechanisms: transstadial, which occurs between life stages; or transovarial, which occurs between mothers and offspring [45]. For one-host ticks such as *R. microplus*, transovarial transmission is one of the most efficient modes of pathogen transfer because ticks spend their entire lifecycle on a single host [46]. As mentioned, *R*. *microplus* ticks are efficient vectors of numerous pathogens, including *B. bovis* (bovine babesiosis), which is passed from the infected females to their offspring [41]. These offspring subsequently seek out and attach to a new host animal, perpetuating the transmission cycle [42]. In the transovarial transmission of *B. bovis*, the pathogen is acquired during adult feeding and moves to the midgut before being released into the hemolymph and migrating to the salivary glands and ovaries. After infecting the ovaries, kinetes are transovarially transmitted to the developing larvae, which then transmit infective sporozoites to naïve hosts via the salivary glands [45]. Successful transovarial transmission, therefore, requires several complex steps, including movement throughout the tick and evading the tick immune system [47]. Unlike *Babesia* sp., the *Theileria* sp. life cycles are not generally characterized by transovarial transmission [48]. The findings in this study are in line with this observation. We assessed ~40,000 pooled larval progeny from each tick group for *T. orientalis* and were unable to detect the parasite. Although the pooling of larvae allowed us to assay a larger percentage of the tick progeny for *T. orientalis*, it also presented a limitation as the separate assessment of larvae from individual female ticks can also provide important information. While the present data suggest a low to non-existent transovarial transmission rate (defined as the number of ticks that produce infected offspring), it is possible that rare, individual females could have a high filial infection rate (defined as the number of infected offspring produced by a single infected female). Essentially, in this case, the overall low transovarial transmission rate could have led to a dilution effect, masking rare females with a high filial infection rate. Future studies in which the offspring of individual females are tested are required to rule out this possibility.

Transstadial transmission is the most common mode of pathogen transfer for most ticks and tick-borne pathogens, including *Theileria* sp. [48,49] This mode of transmission works well for three-host ticks, such as *Haemaphysalis longicornis*, the most common vector of *T. orientalis*. In this system, *T. orientalis* Ikeda is acquired by larval or nymphal *H. longicornis* ticks during a blood meal. Once replete, the tick drops off the host and molts, during this period of off-host development the parasite is passed transstadially to the next stage [2,17,18]. Most tick species that transmit *Theileria* sp. are multi-host ticks and primarily transmit parasites transstadially [45,48]. This major life history difference may explain why *R. microplus* did not transmit *T. orientalis* in our study.

One interesting exception to this general rule is the role of *R. microplus* ticks in transmitting *Theileria equi* between infected horses. *Rhipicephalus microplus* can serve as a tick vector of *T. equi* in some cases [30,43,44]. It has been shown that *T. equi* can be transmitted transstadially from larval or nymphal *R. microplus* to adults [43,50], and that the parasite can be transmitted intrastadially between horses by *R. microplus* adult male ticks, which move between hosts, feeding intermittently, and mating with female ticks [44]. In these studies, the successful *R. microplus* acquisition of *T. equi* from persistently infected horses was demonstrated by the PCR-based detection of the parasite within the salivary gland tissue [43,44,50]. In contrast, we were unable to demonstrate *T. orientalis* within the salivary gland tissue of male or female adult ticks fed on a *T. orientalis*-infected calf. Similarly, while adult *R. microplus* transmits *T. equi* to naïve horses, *R. microplus* was unable to transmit *T. orientalis* to naïve calves.

The definitive reason that *R. microplus* is an efficient vector of *T. equi* but did not acquire or transmit *T. orientalis* in the present study is not clear. We utilized splenectomized calves in this study to maximize parasitemia and minimize the risk of false negative findings, but it is possible that the level of parasitemia to which the ticks were exposed during acquisition (0–0.1%) was insufficient for tick infection. However, in our previous *T. orientalis* Ikeda competency study using the same parasite strain and U.S. *H. longicornis* ticks, the *H. longicornis* ticks successfully acquired and transmitted the parasite when exposed to parasitemia of 0% in a PCR-positive calf [17], and the related parasite *T. parva* was readily transmitted between mammalian hosts by competent tick vectors when the parasite was below the level of detection by light microscopy [51]. Finally, *R. microplus* ticks successfully acquired *T. equi* from persistently infected horses with low-levels of parasitemia [43], suggesting that the parasite dose was not the main variable in play regarding the *R. microplus* competence for *Theileria* sp. acquisition and transmission. Regardless, it remains possible that *R. microplus* could acquire and/or transmit *T. orientalis* under field conditions in which cattle are highly tick-infested and develop extremely high parasitemia.

While this experiment demonstrated that *R. microplus* ticks are not likely competent vectors of *T. orientalis* Ikeda, *T. orientalis* DNA has been detected in *R. microplus* from other regions such as India [33,36], China [34,35], Vietnam [38], and Pakistan [37]. It should be noted that these studies tested field-collected *R. microplus* ticks for *T. orientalis* Ikeda while this experiment utilized colony ticks from Texas. Thus, there could be genetic, behavioral, or physiological differences in the lineages of *R. microplus* from these different regions, or differences in the *T. orientalis* parasite strains [52,53]. Furthermore, detection within a tick does not confirm vector competence unless additional steps to establish competency are conducted. In this controlled experiment, the lack of *R. microplus* vector competence is supported by three main findings: 1. *T. orientalis* was not detected via PCR in the salivary glands from adult ticks fed as larvae and nymphs on a known *T. orientalis*-positive calf; 2. *T. orientalis* was not detected via PCR in the larval progeny of ticks fed on a known *T. orientalis*-infected calf; and 3. No evidence of transmission was detected via PCR, cytology, or clinical pathology in either splenectomized, naïve calves after 60 days. To the authors’ knowledge, this is the first controlled acquisition and transmission study conducted to determine whether *R. microplus* is a competent vector of *T. orientalis* Ikeda.

As a one-host tick, *R. microplus* ticks live on the same host through their three major life stages. With a large percentage of its time spent on the host, it is probable that *R. microplus* will encounter other ticks feeding on the same host. For example, *R. microplus* and *H. longicornis* readily feed on livestock [18,19,20,21,54]. In Tengchong County, China, researchers found both species of ticks feeding on goats and both species were PCR-positive for *T. orientalis* [34]. While the significance of this result is unclear given the results of the present study, other tick-pathogen systems have demonstrated that pathogen spillover from a native tick species into a new tick species is possible. In Virginia, larval, nymphal, and adult stages of *H. longicornis* were positive for the Bourbon virus, a virus that is known to be carried by the lone star tick (*Amblyomma americanum*) [55]. *Amblyomma americanum* ticks and blood from locally harvested deer at the same site also tested positive for the Bourbon virus, suggesting that the tick-borne virus was circulating in the region and that *H. longicornis* could have been infected by feeding on a host that was infected with the Bourbon virus, presumably infected by *A. americanum* [55]. Pathogen spillover into a new tick host can lead to active pathogen circulation in the absence of the known primary tick vector. Through surveillance and experimental studies, pathogen spillover from a native tick species to a new tick species should be prioritized as a future research avenue to predict the range expansion of tick-borne pathogens. This is especially relevant to *T. orientalis*, which is newly endemic in the U.S.

Co-feeding next to an infected tick has also been suggested as a possible route of pathogen sharing between ticks. The primary vector for *Rickettsia parkeri* is the Gulf Coast tick, *Amblyomma maculatum* [56]. However, when *A. americanum* nymphs were co-feeding next to infected *A. maculatum*, the pathogen was successfully transstadially maintained in *A. americanum* adults [56]. Feeding in close spatiotemporal proximity has been illustrated as an efficient way to transmit pathogens between two ticks in the absence of systemic pathogen infection in a host [57,58]. While *T. orientalis* may not efficiently infect the *R. microplus* ticks in the absence of co-feeding *H. longicornis* ticks, further studies are needed to rule out *Theileria* sp. Transmission between ticks via the close proximity feeding of two different tick species.

*T. orientalis* will likely continue to cause significant losses for the U.S. cattle industry. The extent of the *T. orientalis* spread and the resultant development of regional endemicity is dependent both on the movement of asymptomatic carrier cattle and the range of competent tick hosts. Many tick species native to the U.S. are vectors of various *Theileria* sp. parasites. Thus, continued studies are needed to characterize the repertoire of tick vectors for this newly endemic pathogen of U.S. cattle. While the present study suggests that *R. microplus* is not likely a competent vector for *T. orientalis*, surveillance is recommended to monitor for interactions between *H. longicornis*, *R. microplus*, and other native U.S. ticks to ensure that pathogen spillover does not occur.

## Figures and Tables

**Table 1:** Weekly *Theileria orientalis* peripheral blood PCR results for Calves 1–3. DPI = days post-infection. During weeks 3, 5, and 11, *T. orientalis* was below the level of detection for at least 1 sampling day that week but was detected on other sampling points in the same week. This is indicated with +/−.

Calf	1 0–7 DPI	2 8–14 DPI	3 15–21 DPI	4 22–28 DPI	5 29–35 DPI	6 36–42 DPI	7 43–49 DPI	8 50–56 DPI	9 57–63 DPI	10 64–70 DPI	11 71–77 DPI	12 78–84 DPI	13 85–91 DPI	14 92–98 DPI	15 99–105 DPI	16 106–112 DPI	17 113–119 DPI
**1**	−	−	+/−	+	+/−	+	+	+	+	+	+/−	+	+	+	+	+	+
**2**	−	−	−	−	−	−	−	−	−	−	−	−	−	−	−	−	−
**3**	−	−	−	−	−	−	−	−	−	−	−	−	−	−	−	−	−

**Table 2:** Results of *Theileria orientalis* PCR performed on adult *Rhipicephalus microplus* salivary glands and macerated *R. microplus* larvae.

Tick Batch	Females Tested	Males Tested	Number Positive
**Group 1 Adults**	6	9	0
**Group 2 Adults**	7	8	0
**Group 1 Larvae**	~20,000 larval ticks		Batch negative
**Group 2 Larvae**	~20,000 larval ticks		Batch negative

## Data Availability

All data are provided in the manuscript.
